# Harnessing accurate mitochondrial DNA base editing mediated by DdCBEs in a predictable manner

**DOI:** 10.3389/fbioe.2024.1372211

**Published:** 2024-04-09

**Authors:** Jiaxin Qiu, Haibo Wu, Qin Xie, Yuxiao Zhou, Yining Gao, Junbo Liu, Xueyi Jiang, Lun Suo, Yanping Kuang

**Affiliations:** Department of Assisted Reproduction, Shanghai Ninth People’s Hospital, Shanghai Jiao Tong University School of Medicine, Shanghai, China

**Keywords:** mitochondrial genome, mtDNA, gene editing, DdCBEs, predictability

## Abstract

**Introduction:** Mitochondrial diseases caused by mtDNA have no effective cures. Recently developed DddA-derived cytosine base editors (DdCBEs) have potential therapeutic implications in rescuing the mtDNA mutations. However, the performance of DdCBEs relies on designing different targets or improving combinations of split-DddA halves and orientations, lacking knowledge of predicting the results before its application.

**Methods:** A series of DdCBE pairs for wide ranges of aC or tC targets was constructed, and transfected into Neuro-2a cells. The mutation rate of targets was compared to figure out the potential editing rules.

**Results:** It is found that DdCBEs mediated mtDNA editing is predictable: 1) aC targets have a concentrated editing window for mtDNA editing in comparison with tC targets, which at 5’C_8-11_ (G1333) and 5’C_10-13_ (G1397) for aC target, while 5’C_4-13_ (G1333) and 5’C_5-14_ (G1397) for tC target with 16bp spacer. 2) G1333 mediated C>T conversion at aC targets in DddA-half-specific manner, while G1333 and G1397 mediated C>T conversion are DddA-half-prefer separately for tC and aC targets. 3) The nucleotide adjacent to the 3’ end of aC motif affects mtDNA editing. Finally, by the guidance of these rules, a cell model harboring a pathogenic mtDNA mutation was constructed with high efficiency and no bystander effects.

**Discussion:** In summary, this discovery helps us conceive the optimal strategy for accurate mtDNA editing, avoiding time- and effort-consuming optimized screening jobs.

## Introduction

The mitochondrial genome is very small, including only 37 genes encoding 2 ribosomal RNAs, 22 transfer RNAs, and 13 proteins, but it is essential for cells to produce energy through oxidative phosphorylation, and a single-nucleotide mutation in a specific region of mitochondrial DNA (mtDNA) could result in severe metabolic disorders in humans (Schapira, 2012; Gorman et al., 2016; Grady et al., 2018; Meng et al., 2022). Since mitochondrial diseases mainly affect multiple high energy-demanded organs, these disorders are characterized by serious disability or even fatality (McFarland et al., 2010), which consumes greater care and economic support both from the family and the society. Most importantly, there are extremely limited licensed cures available thus far for this series of disabling or life-limiting disorders (Chinnery, 2015; Viscomi et al., 2023). Treatment methods for mitochondrial diseases include symptomatic treatments to improve quality of life or increase life expectancy, and gene therapy to decrease heteroplasmy and cure the cellular biochemical defect. Symptomatic treatments include manipulating cell content of mitochondria, inducing mitochondrial turnover through rapamycin, restoring NAD^+^ levels, modulating the production of reactive oxygen species and oxidative stress, ect (Russell et al., 2020). Gene therapy includes direct editing of mitochondrial genomes, gene replacement therapy (Silva-Pinheiro et al., 2020; Ling et al., 2021), and mitochondria transfer therapy (Greenfield et al., 2017).

Gene editing techniques, acting as a potential therapeutic option, have been widely investigated in treatment of the nuclear genetic diseases over the past decade (Sharma et al., 2015; Nelson et al., 2016; De Ravin et al., 2017; Zheng et al., 2022), with an increasing number of clinical trials ongoing (Arabi et al., 2022). However, its implication in mitochondrial diseases caused by mtDNA mutations has been hampered by the lack of efficient tools to manipulate the mtDNA (Silva-Pinheiro and Minczuk, 2022), with the exception that deleterious mtDNA copies could be cut and eliminated by Zinc finger-fused (Minczuk et al., 2008; Gammage et al., 2014; Gammage et al., 2016a; Gammage et al., 2016b; Gammage et al., 2018b) or TALE-fused fokI nuclease (Bacman et al., 2013; Reddy et al., 2015; Bacman et al., 2018; Pereira et al., 2018; Yang et al., 2018), as well as the monomeric enzyme based on the TALE system (Pereira et al., 2018).

Till recently, TALE-based mtDNA base editing tools have been introduced, and the first one was DddA-derived cytosine base editors (DdCBEs) (Mok et al., 2020), which open the doors for manipulating mtDNA as intended. DddA system is derived from Burkholderia, and DdCBEs are composed of two non-toxic halves of TALE fused split-DddA (DddA-N and DddA-C) and catalyze the deamination of cytidines within the spacing region via reassembly of these two split DddA halves to a functional deaminase. At present, DdCBEs have been successfully applied for mtDNA editing in plants (Kang et al., 2021), mammalian cells (Mok et al., 2020), zebrafish (Guo et al., 2021), mice (Lee et al., 2021; Lee et al., 2022a; Guo et al., 2022), rats (Qi et al., 2021), and even human germ cells (Wei et al., 2022a; Chen et al., 2022). In our lab, it has also successfully been used for efficient germline mtDNA editing at the early follicular stage in mice (submitted data). Unfortunately, its application in rescuing mitochondrial diseases is extremely rare, either for therapeutic investigation (Silva-Pinheiro et al., 2022) or for clinical trials (Chen and Yu-Wai-Man, 2022).

It is known that the predictability of the potential gene editing results is critical for gene editing techniques to be used for gene therapy in clinics. For this purpose, a great many works have been done to understand the edit rules of the CRISPR system for different targets in nuclear genome editing, and it has been demonstrated that the consequence is completely predictable for each protospacer to be edited by CRISPR/Cas9 (van Overbeek et al., 2016; Shen et al., 2018; Shou et al., 2018; Allen et al., 2019; Chakrabarti et al., 2019; Chen et al., 2019; Long, 2019; Shi et al., 2019), which allow us to know the potential outcomes in advance for each strategy to be used in clinics. However, for the mitochondrial genome, CRISPR/Cas9 has not been implied in the mtDNA editing owing to the lack of DNA repair pathway mediated by homology-directed repair (HDR) and non-homologous end-joining (NHEJ) in the mitochondrial genome, and the linearized mitochondrial DNA would be rapidly degraded and eliminated (Nissanka et al., 2018; Peeva et al., 2018). Moreover, base editors based on the CRISPR system have also been hindered in mtDNA editing as it relies on the unwinding of double-strand DNA mediated by guide RNA, but unfortunately, guide RNAs could not be introduced into the mitochondrial matrix (Gammage et al., 2018a).

As an alternative, TALE-based DdCBE is an all-protein base editor, which could immediately catalyze the deamination of cytidines within double-strand DNA. It has been reported that DdCBEs strongly prefer 5′-tC targets or even 5′-aC targets for mtDNA editing (Boyne et al., 2022; Qi et al., 2023; Silva-Pinheiro et al., 2023), although some labs have expanded the scope of the target by constructing some other DddA variants (Mok et al., 2022; Guo et al., 2023; Mi et al., 2023; Wei et al., 2023). Meanwhile, based on DdCBE, a series of related variants have been developed one after another, including DddAtox mutant-containing DdCBE with higher editing efficiency and broader editing scope (Mok et al., 2022), transcription-activator-like effector-linked deaminases (TALEDs) which can introduce A-G substitution (Cho et al., 2022) and strand-selective base editors (mitoBEs) with high A-G editing efficiency and specificity (Yi et al., 2023), as well as strand-preferred base editor (CyDENT) with high strand-specificity and broader editing scope (Hu et al., 2023). Despite these variants, DdCBE still acts as the most commonly used editor for mtDNA. But the understanding of the editing rules is very limited, especially for the aC targets. It is urgently needed to optimize the editing strategies by adjusting the compatibility of DddA splits with different TALE designs and deaminase orientations before its application.

In this work, by screening the combination of different DdCBE pairs for a wide range of tested mtDNA targets, it is found that DdCBE edits mtDNA are predictable. This finding will guide us to perform mtDNA editing accurately and without the bystander effects, avoiding time- and effort-consuming screening jobs for strategy optimization before its application.

## Material and methods

### Construction of TALE-fused DdCBEs

The DdCBE vectors used were synthesized in Sangon Biotech (Shanghai), which is composed of mitochondrial localization sequence (MTS), N terminal, C terminal, one kind of four split DddA halves (N terminal split at G1333 (G1333-N), C terminal split at G1333 (G1333-C), N terminal split at G1397 (G1397-N), and C terminal split at G1397 (G1397-C)), and UGI-coding sequences. Four pairs were designed for each site according to the two different splits with two different orientations (G1333CL+G1333NR, G1397CL+G1397NR, G1333NL+G1333CR, and G1397NL+G1397CR). G1333CL+G1333NR pair consists of G1333C fused to left TALE array (G1333CL) and G1333N fused to right TALE array (G1333NR); G1397CL+G1397NR pair consists of G1397C fused to left TALE array (G1397CL) and G1397N fused to right TALE array (G1397NR); G1333NL+G1333CR pair consists of G1333N fused to left TALE array (G1333NL) and G1333C fused to right TALE array (G1333CR); G1397NL+G1397CR pair consists of G1397N fused to left TALE array (G1397NL) and G1397C fused to right TALE array (G1397CR). TALE arrays were constructed using Golden Gate TALEN and TAL Effector Kit 2.0 (Addgene). The corresponding protein sequences are listed in the Supplementary Table S1. The Repeat Variable Diresidues (RVDs) containing NI, NG, NN and HD amino acids, recognize A, T, G and C, respectively. Ligated plasmids were transformed into Trans5α chemically competent cells (TransGene Biotech) and subjected to Sanger sequencing to analyze the identity of the constructs (Sangon Biotech). Final plasmids were prepared using Endofree mini plasmid kit II (TianGen) for cell transfection.

### Cell culture and transfection

Neuro-2a cells (CCL-131; ATCC) were cultured in MEM (BasalMedia) with 10% fetal bovine serum (FBS, Gibco), 1% sodium pyruvate (Introvigen), 1% Non-Essential Amino Acids (NEAA, Gibco) and 1% penicillin–streptomycin (Gibco) at 37°C with 5% CO_2_. U2-OS cells (HTB-96; ATCC) were cultured in high-glucose DMEM (Gibco)+10% FBS+1% penicillin–streptomycin at 37°C with 5% CO_2_.

For transfection, Neuro-2a cells were plated in 24-well cell culture plates at a density of 2.4 × 10^5^ per well. After 24 h, lipofection was performed at a cell density of approximately 70%. Cells were transfected with 800 ng plasmid of each mitoTALE monomer. 0.75 μL LipofectamineTM 3000 Reagent and 1 μL P3000TM Reagent were used per well. 24 h after transfection, the original culture medium was replaced with a medium containing 1 mg/mL G418 (MCE). Cells were collected after screening for 72 h. U2-OS cells were plated in 12-well cell culture plates at a density of 3 × 10^5^ per well 24 h before lipofection. Cells were transfected with 1,600 ng plasmid of each mitoTALE monomer, using 1.5 μL LipofectamineTM 3000 Reagent and 2 μL P3000TM Reagent per well. G418 (1 mg/mL) screening starts 24 h after transfection and lasts for 5.5 days. Genomic DNA was extracted using the DNeasy Blood and Tissue Kit (Qiagen) and stored at −20°C till sequence library construction. 

### Sanger sequencing and next-generation sequencing (NGS)

Sanger sequencing was performed by Sangon Biotech (Shanghai). The primers for each spacer are listed in Supplementary Table S2. For NGS, Phanta Flash DNA Polymerase (Vazyme) was used to amplify target sequences with primers containing barcodes and Illumina adapters in the first round PCR (100 ng genome DNA extracted from cells as template). The sequence of primers is listed in Supplementary Table S2. PCR product of the first round was used for the second round of PCR using index primers (Vazyme). After the second round of PCR, samples with different barcodes and indexes were mixed, purified by gel extraction using the QIAquick Gel Extraction Kit (Qiagen), and quantified using the Qubit ssDNA HS Assay Kit (Thermo Fisher Scientific). Deep sequencing was performed on the Illumina NovaSeq 6000 platform. Quality control was performed for the sequencing data by fastp (v0.23.2) using default parameters. The sequencing reads were demultiplexed using fastq-multx (v1.4.1) with the barcoded PCR primers, and the editing frequencies of the on-target site were calculated by output file from batch analysis with CRISPResso2 (v2.0.32), and statistics were generated using in-house scripts with R (v4.2.1).

### Whole mtDNA sequencing

The whole mtDNA was amplified as two overlapping 8 kb fragments by long-range PCR, and the sequence information of the primers was shown in Supplementary Table S2. The PCR products were purified by QIAquick Gel Extraction Kit (Qiagen) and used as input for constructing libraries using TruePrepTM DNA Library Prep Kit V2 for Illumina (Vazyme). The libraries were purified using DNA clean beads and quantified using the Qubit ssDNA HS Assay Kit (Thermo Fisher Scientific) before performing the deep sequencing. To analyze NGS data from whole mitochondrial genome sequencing, the qualified reads were mapped to the mouse mitochondrial reference genome (mm10) by BWA (v0.7.12) with mem–M, and then generated BAM files with SAMtools (v.1.9). Positions with conversion rates ≥0.1% were identified among all cytosines and guanines in the mitochondrial genome using the REDItoolDenovo.py script from REDItools (v.1.2.1).

### Calculation of average off-target editing frequency

Single-nucleotide variants present in both treated and untreated samples (that therefore did not arise from DdCBE treatment) were excluded. The average off-target editing frequency was then calculated independently for each biological replicate of each treatment condition as: (number of reads in which a given C•G base pair was called as a T•A base pair, summed over all non-target C•G base pairs)/(total number of reads that covered all non-target C•G base pair).

### Statistical analysis

Figures were drawn with GraphPad Prism 9, Figdraw (www.figdraw.com), and Adobe Illustration 2021. Mean and standard error of measurement (SEM) of editing efficiency of three biological duplicate samples was calculated using SPSS software (version 23.0; SPSS Inc., Chicago, IL, USA). Independent sample *t*-test was applied to comparison of normally distributed data (ns *p* > 0.05, *p < 0.05, **p < 0.01, ***p < 0.001, ****p < 0.0001).

## Results

### The aC targets have a more concentrated editing window than tC targets

**FIGURE 1:**
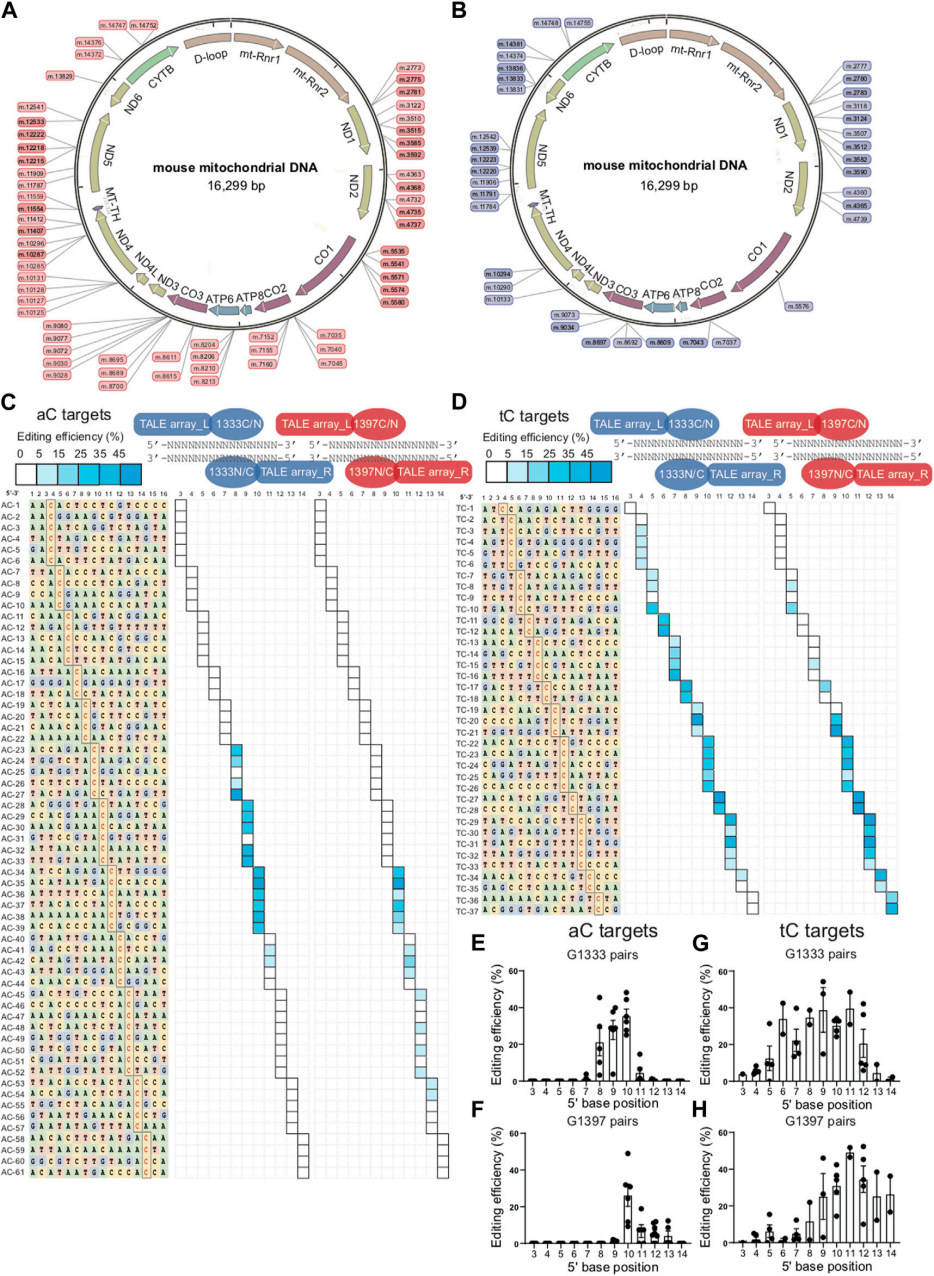
Editing window of aC and tC targets for DdCBE mediated mtDNA editing. **(A, B)** Genetic map of mouse mtDNA indicating the selected aC or tC targets located in the mitochondrial genome. aC targets were marked in pink and tC targets were marked in purple. **(C)** Base information of all 61 aC target spacers and the targeting cytosine is highlighted in red (left panel). The heat map showing the efficiency of C>T conversion at aC targets with optimal pairs of corresponding splits of DddA pairs (G1333 in the middle panel and G1397 in the right panel). The shading levels of solid squares in the map indicated the mean editing values from three independent biological replicates for each aC target site. **(D)** Base information of all 37 tC target spacers and the targeting cytosine is highlighted in red (left panel). The heat map showing the efficiency of C>T conversion at tC targets with optimal pairs of corresponding splits of DddA pairs (G1333 in the middle panel and G1397 in the right panel). The shading levels of solid squares in the map indicated the mean editing values from three independent biological replicates for each tC target site. **(E)** Mean editing efficiency of G1333 pairs for aC targets with cytosine at different positions (5′C_3-14_) within the 16bp spacer. **(F)** Mean editing efficiency of G1397 pairs for aC targets with cytosine at different positions (5′C_3-14_) within the 16bp spacer. **(G)** Mean editing efficiency of G1333 pairs for tC targets with cytosine at different positions (5′C_3-14_) within the 16bp spacer. **(H)** Mean editing efficiency of G1397 pairs for tC targets with cytosine at different positions (5′C_3-14_) within the 16bp spacer. For e-h, only the editing efficiency of the pair with higher editing efficiency was included for specific split. For **(E–H)**, each dot represents the average editing efficiency of each separate target at the same position (at least three) from Figure **(C, D)**. For each target, three biological replicates were transfected and sequenced independently. The values and error bars reflect the mean ± sem of each separate target.

To explore the editing window of mtDNA targets mediated by DdCBEs, nearly one hundred mtDNA target sites were selected, and the location of each site in the mitochondrial genome was marked in Figure 1A (for aC targets) and Figure 1B (for tC targets). To minimize the variation among different targets, a commonly used spacer length with 16 base pairs was designed for all of the selected sites. For aC editing, a total of 61 sites was designed ensuring that there are at least 3 different aC sites for each position between 5′C_3-14_ in the spacing region (Figure 1C left panel), and the detailed information of each target is shown in Supplementary Table S3. For each spacer, four DdCBE pairs (G1333CL+G1333NR, G1397CL+G1397NR, G1333NL+G1333CR, and G1397NL+G1397CR) were constructed. After four pairs were transfected into the Neuro-2a cells, the efficiency of C>T conversion at each site was evaluated by the next-generation sequencing (NGS) method, and the results indicated that there is a wide range of mtDNA editing efficiencies (from 1.03% ± 0.46% to 48.99% ± 2.05%) depending on the position of the target C within the spacing region (Supplementary Table S4). When the data for the tC targets were also evaluated with a similar strategy (Supplementary Table S5), it was observed that the editable range of aC targets was more concentrated than that of tC targets, either for the G1333 splits of DddA or for the G1397 splits of DddA. The higher editing efficiency of G1333 pairs or G1397 pairs of each aC target was shown in Figure 1C and that of each tC target was shown in Figure 1D, and the specific editor combination used for each site is listed in Supplementary Table S6. The mean efficiency of C>T conversion for each target cytosine at different positions for aC and tC targets is separately shown in Figures 1E–H. Editing windows are defined when there exist targets with editing efficiency >5% in the position. As Figure 1C indicated, the editing window of aC targets could be defined as 5′C_8-11_ for the G1333 split of DddA, while high efficiency (>15%) only could be achieved when the cytosine was put at the position of 5′C_8-10_ in the spacer (Figure 1C middle panel; Figure 1E). If aC targets were edited by the G1397 split of DddA, the editing window was defined as 5′C_10-13_, but the mean editing efficiency was relatively low (4.39% ± 2.56% to 6.75% ± 3.46%) when the cytosine was put at 5′C_11-13_ in the spacer (Figure 1C right panel; Figure 1F). Whereas, when the editing window of the tC targets was evaluated, it was observed that tC targets have a broader editing window than aC targets. As the results showed, tC targets could be efficiently edited when the cytosine was put at 5′C_4-13_ in the spacer for the G1333 split of DddA (Figure 1D middle panel), while average editing efficiency (>15%) only could be achieved in 5′C_6-12_ (Figure 1G). Meanwhile, the editing window for the tC target was between 5′C_5-14_ when it was edited by the G1397 split of DddA (Figure 1D right panel), while the average editing efficiency of more than 15% only could be obtained in 5′C_9-14_ (Figure 1H). In summary, all of the results above indicated that the editing window of aC is more concentrated in comparison with that of tC targets, which means aC editing has stricter requirements for spacer selection.

### G1333 mediated C>T conversion in DddA-half-specific manner, while G1333 and G1397 mediated C>T conversion are just DddA-half-prefer separately for tC and aC targets

It was found the editing effciency between different orientations of the same loci (G1333CL+G1333NR vs. G1333NL+G1333CR, and G1397CL+G1397NR vs. G1397NL+G1397CR) can have a huge difference. Thus it was suspected whether there exited DddA-half-specificity or DddA-half-preference for editing. DddA-half-specificity was defined as the pair can only edit targets on the specific strand, while the pair of the other orientation can not or barely not edit targets on the strand. DddA-half-preference was defined when pairs derived from the same split can both edit effectively for a specific strand but the editing efficiency of one pair is always higher than that of the other pair with different orientation.

**FIGURE 2:**
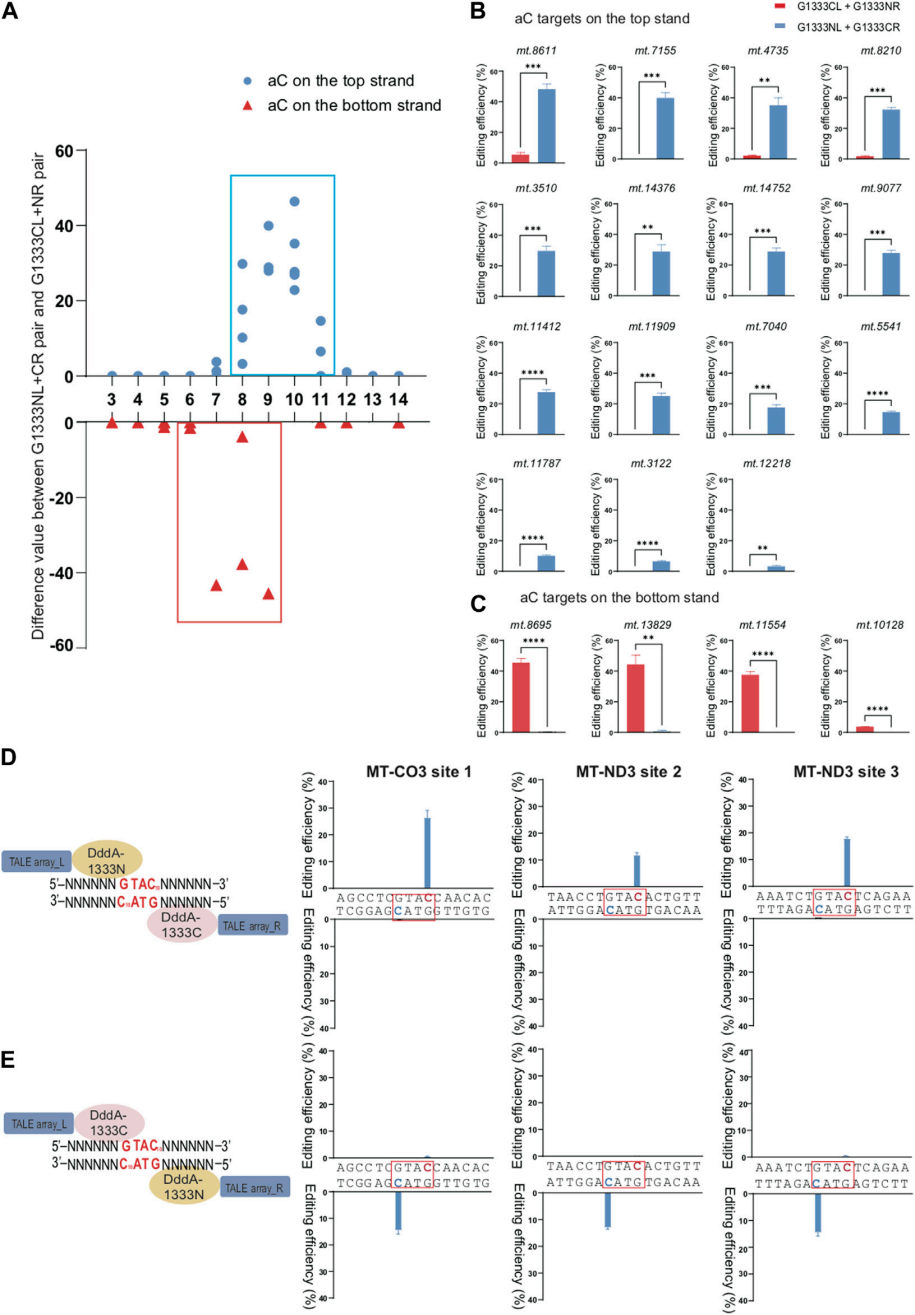
Editing specificity of G1333 split of DddA for aC motif. **(A)** The difference value of aC editing between G1333NL+G1333CR pair and G1333CL+G1333NR pair of each locus. **(B, C)** Comparison of editing efficiency of aC sites on the top strand **(B)** and bottom strand **(C)** using G1333 pair of different orientations. Editing window was framed with blue and red box respectively, and all the aC sites in the editing window were included. (Values and error bars reflect the mean ± sem of n = 3 independent biological replicates. Independent sample *t*-test: **p < 0.01, ***p < 0.001, ****p < 0.0001). **(D)** Schematic models two different orientations of G1333 splits of DddA target the spacer with two aC motifs separately on the top and bottom strands. **(E)** Two-way column indicated the efficiency of C>T conversion of aC motif separately located on the top and bottom strand within MT-CO3 site 1 (m.8928 C>T on the top strand; m.8925 C>T on the bottom strand, left panel), MT-ND3 site 2 (m.9470 C>T on the top strand; m.9467 C>T on the bottom strand, middle panel) and MT-ND3 site 3 (m.9548 C>T on the top strand; m.9545 C>T on the bottom strand, right panel) when they were edited by G1333NL+G1333CR pair and G1333CL+G1333NR pair, respectively. Target cytosine are highlighted in red and green respectively for the top and bottom strands.

To compare the effect of orientations on the editing efficiency for specific splits of DddA separately to edit aC and tC targets, we used the difference value of editing efficiency between G1333NL+G1333CR pair and G1333CL+G1333NR pair as the vertical axis for G1333 split of DddA, while the difference value of editing efficiency between G1397CL+G1397NR pair and G1397NL+G1397CR pair as the vertical axis for G1397 split of DddA. As Figure 2A indicated, if this value is positive, it means that the editing efficiency of G1333NL+G1333CR pair is higher than G1333CL+G1333NR pair, while if this value is negative, it means that the editing efficiency of G1333NL+G1333CR pair is lower than G1333CL+G1333NR pair. By using this method, it was observed that when G1333 splits of DddA were used for aC editing, the cytosine of aC motif in the top strand can only be edited by G1333NL+G1333CR pair, whereas it almost cannot be edited when the G1333CL+G1333NR pair was used (Figures 2A, B). Remarkably, the case was the opposite when aC motif was in the bottom strand (Figures 2A, C). Herein, it was speculated that G1333 split of DddA might edit aC targets in a half-specific manner. To verify this hypothesis, three additional targets (MT-CO3 site 1; MT-ND3 site 2; MT-ND3 site 3) were selected, and each target has two aC motifs separately located at the top and bottom strands in the same position (the 10th nucleotide) from the 5′ ends of each strand (Figure 2D). When the editing efficiency was evaluated in three different sites, it was also observed that the editing only occurred in the aC motif on the top strand when using G1333NL+G1333CR pair, whereas editing only occurred in the aC motif on the bottom strand when switching the combination (G1333CL+G1333NR pair). Remarkably, these results were perfectly reproducible both in MT-CO3 site 1 (Figure 2E left panel), MT-ND3 site 2 (Figure 2E middle panel) and MT-ND3 site 3 (Figure 2E right panel).

**FIGURE 3:**
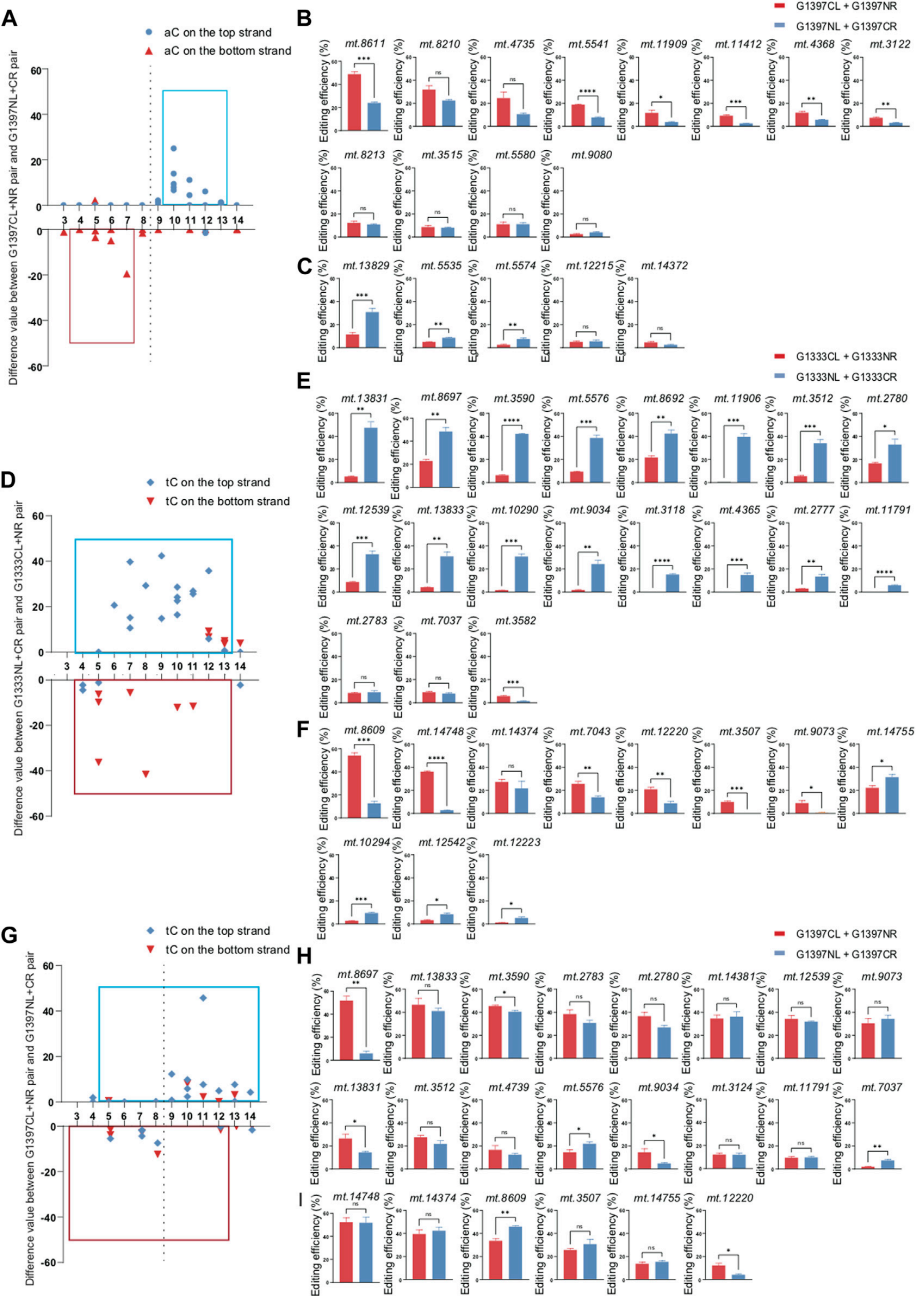
Editing efficiency of different DdCBE pairs for aC or tC motif at the top and bottom strand. **(A)** The difference value of aC editing between G1397CL+G1397NR pair and G1397NL+G1397CR pair for each locus. **(B, C)** Comparison of editing efficiency of aC sites on the top strand **(B)** and bottom strand **(C)** using G1397 pair of different orientations. **(D)** The difference value of tC editing between G1333NL+G1333CR pair and G1333CL+G1333NR pair for each locus. **(E, F)** Comparison of editing efficiency of tC sites on the top strand **(E)** and bottom strand **(F)** using G1333 pair of different orientations. **(G)** The difference value of tC editing between G1397CL+G1397NR pair and G1397NL+G1397CR pair for each locus. **(H, I)** Comparison of editing efficiency of tC sites on the top strand **(H)** and bottom strand **(I)** using G1397 pair of different orientations. Editing window was framed with blue and red box respectively, and all of the editable sites in the marked editing window were included. (Values and error bars reflect the mean ± sem of *n* = 3 independent biological replicates. Independent sample *t*-test: *p < 0.05, **p < 0.01, ***p < 0.001, ****p < 0.0001).

Different from G1333 splits of DddA, both orientations of G1397 splits can edit the aC sites (Figure 3A). To further explore whether the editing scope of different orientations had a preference, we compared the difference value between G1397CL+G1397NR pair and G1397NL+G1397CR pair at different loci (Figures 3B, C). It was found that both orientations could work, but G1397CL+G1397NR pair had significantly higher editing efficiency when the aC motif was on the top strand for most of the selected targets. Oppositely, most of the aC motif on the bottom strand could be edited more effectively by the G1397NL+G1397CR pair. Notably, as Figure 3A indicated, the editable cytosine was mainly concentrated on the right half of the spacing region.

For tC targets, the half-preference only occurred for G1333 but not G1397 splits of DddA (Figures 3D, G). As the results indicated, most of the G1333NL+G1333CR pair had higher editing efficiency when the tC motif in the top strand was edited, while most of the G1333CL+G1333NR pair worked better when the tC motif in the bottom strand was edited (Figures 3E, F). Remarkably, there is no obvious strand bias exists for the G1397 splits of DddA (Figure 3G). No matter which strands the tC motif is located in, both orientations of the G1397 splits of DddA pairs have certain levels of editing efficiency (Figures 3H, I). Interestingly, when calculating the difference value between G1397CL+G1397NR pair and G1397NL+G1397CR pair for all loci (Figure 3G), it was found that tC on the right half of the spacer was edited with priority by the G1397CL+G1397NR pair; while the G1397NL+1397CR pair preferentially edited the tC on the left half of the spacer. Taken together, G1333 split of DddA mediated aC and tC editing separately in half-specific and half-prefer manner, while half-prefer only exists for G1397 split of DddA when it was used for editing aC targets.

### The nucleotide adjacent to the 3′ end of aC motif affects the efficiency of mtDNA editing

**FIGURE 4:**
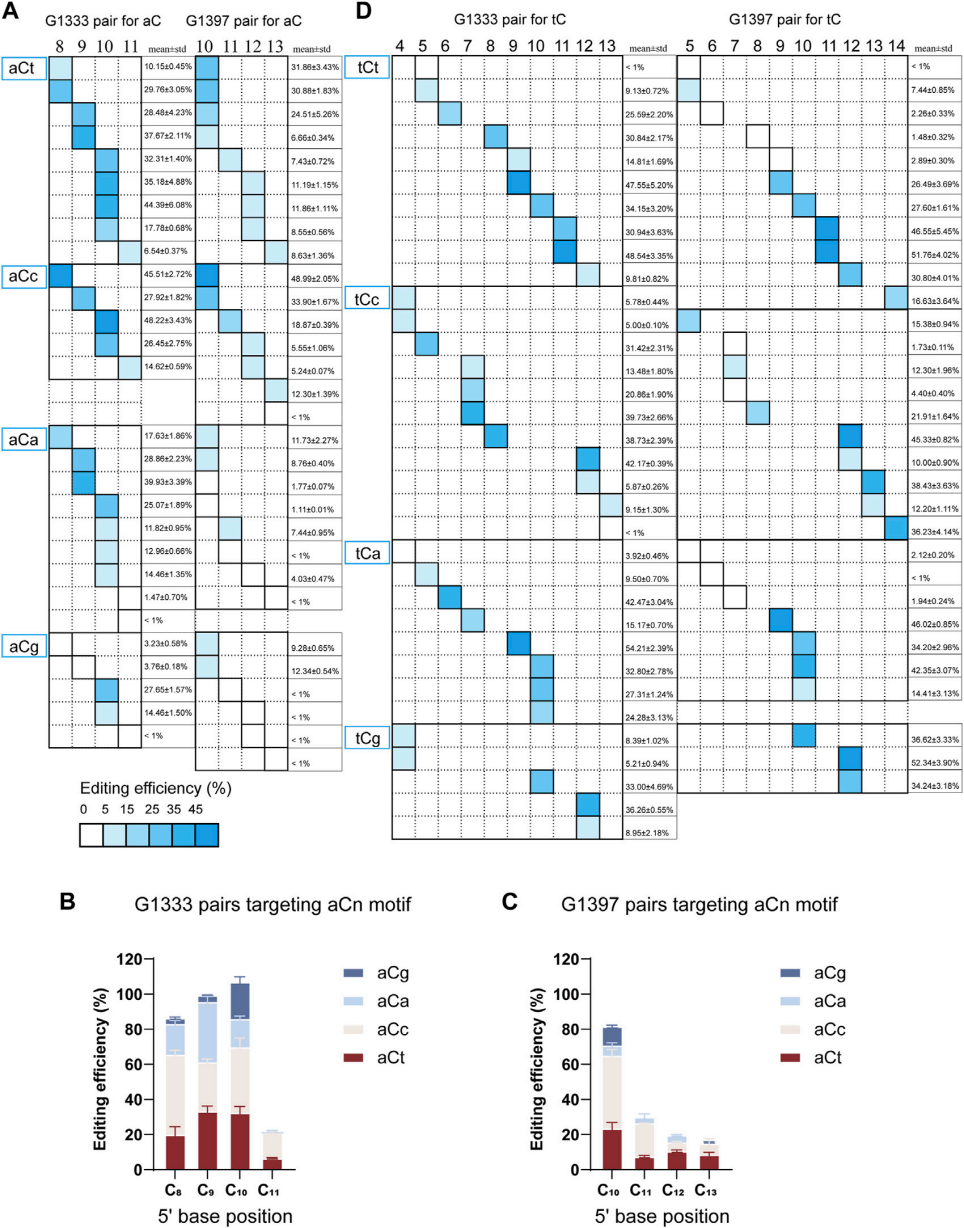
Effect of nucleotide adjacent to the 3′ end of aC motif on the mtDNA editing. **(A)** The heat map showing the efficiency of C>T conversion for the aCn (*n* = t, c, a, g) motif at different positions (5′C_8-11_ for G1333 splits and 5′C_10-13_ for G1397 splits) within the spacer by G1333 splits of DddA (left panel) and G1397 splits of DddA (right panel). The mean ± sem of *n* = 3 independent biological replicates were shown behind the squares. **(B, C)** The column compared the editing efficiency aC motif at the same position within the spacer with different nucleotides adjacent to 3′end when it was edited by G1333 splits of DddA pairs **(B)** and G1397 splits of DddA pairs **(C)**. **(D)** The heat map showing the efficiency of C>T conversion for the tCn (*n* = t, c, a, g) motif at different positions (5′C_4-13_ for G1333 splits and 5′C_5-14_ for G1397 splits) within the spacer by G1333 splits of DddA (left panel) and G1397 splits of DddA (right panel). The shading levels of the solid squares in the map indicated the mean editing value from three independent biological replicates for the optimal pair of each split of DddA. The mean ± sem of *n* = 3 independent biological replicates were shown behind the squares.

According to the results from editing windows screening, it was observed that some sites are difficult to edit even if they are located within the editing window. Thus it was speculated that the nucleotide adjacent to the 3′ end of aC motif might impact the mtDNA editing results. To further confirm our hypothesis, all the aCn motifs in the editing window were classified based on the nucleotide adjacent to the 3′ end (n for t, c, a, or g), and screened mtDNA editing with the different combinations of DdCBE pairs. For each site, all the four DdCBE pairs were constructed and tested. And the editing efficiency of the pair having better editing results of the same split method was used in Figure 4. It was observed that DdCBEs catalyzed the C>T conversion in a motif-dependent manner, and the motif with aCt and aCc can be edited throughout the entire editing windows (5′C_8-11_ for the G1333 split and 5′C_10-13_ for the G1397 split) (Figure 4A). For the motif with aCa, effective editing (>15%) can only be achieved when placing the target at 5′C_8-10_ using the G1333 splits (Figure 4A). As the results summarized in Figures 4B, C, aCt and aCc can achieve relatively higher editing efficiency than aCa and aCg in different positions regardless of the editor combinations, aCa motif could be edited effectively with G1333 splits of DddA only when the cytosine was put at the 5′C_8-10_, and the aCg motif could only be edited when the cytosine was put at the 5′C_10_ in the spacing region either for G1333 or for G1397 splits.

When further exploring the influence of the 3′ end nucleotide on the tC editing, it was found there is no significant association between the specific motif and editing efficiency. According to the currently limited data, the base propensity was not applicable for tC editing, because tC motif with different adjacent nucleotides at the 3′ end (tCa/tCt/tCc/tCg) all work comparably (Figure 4D) for both G1333 and G1397 pairs.

### The length of the spacing region affects the position of editable cytosine for aC targets

**FIGURE 5:**
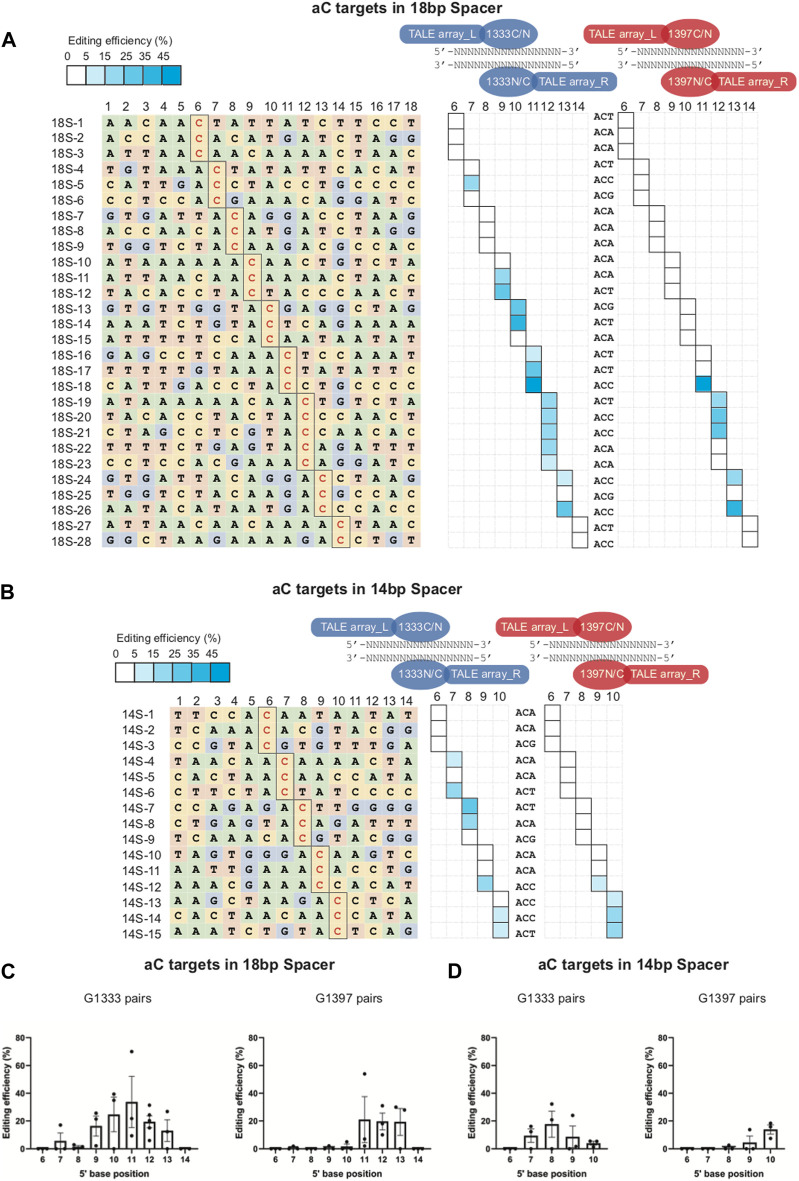
Editing window of aC targets within 18bp and 14bp spacer. **(A)** Base information of aC target with 18bp spacer and the targeting cytosine is highlighted in red (left panel). The heat map showing the efficiency of C>T conversion at aC targets with optimal pairs of corresponding splits of DddA pairs (G1333 in the middle panel and G1397 in the right panel). **(B)** Base information of aC target with 14bp spacer and the targeting cytosine is highlighted in red (left panel). The heat map showing the efficiency of C>T conversion at aC targets with optimal pairs of corresponding splits of DddA pairs (G1333 in the middle panel and G1397 in the right panel). The shading levels of solid squares in the map indicated the mean editing values from three independent biological replicates for each aC target site. **(C)** Mean editing efficiency of the different aC targets (5’C_6-14_) within the 18bp spacer when they were edited by the G1333 split and G1397 split, respectively. **(D)** Mean editing efficiency of the different aC targets (5’C_6-10_) within the 14bp spacer when they were edited by the G1333 split and G1397 split, respectively. For C-D, only the editing efficiency of the pair with higher editing efficiency for each split of DddA was included, and the editing efficiency was calculated by the average efficiency of different locus at the same position, and values and error bars reflect the mean ± sem.

It is known that, due to the restrictions of TALEN design, the length of the spacing region is variable rather than fixed when performing the mtDNA editing in practice. To further investigate the editing rules of the aC targets when the spacer length is more or less than 16 base pairs, some additional aC targets with 18bp (Figure 5A left panel) and 14bp (Figure 5B left panel) in the spacing region were selected for mtDNA editing, and the detailed information of these targets was shown in Supplementary Table S3). For the 18bp spacer, it was found the editing window of G1333 pairs was wider as the spacer length increased – 5′C_7-13_, compared with 5′C_8-11_ of the 16bp spacer (Figure 5A middle panel; Figure 5C). The editing window of G1397 pairs was between 5′C_11-13_ for the 18bp spacer compared with 5′C_10-13_ for the 16bp spacer (Figure 5A right panel; Figure 5D). Correspondingly, for the 14bp spacer, the editing window of G1333 pairs was relatively shorter -5′C_7-10_ (Figure 5B middle panel; Figure 5E), while aC can be edited by G1397 splits of DddA only when the cytosine was put at 5′C_9_ and 5′C_10_ (Figure 5B right panel; Figure 5F). Remarkably, there were also exists some uneditable sites within the editing window both for the 18bp and the 14bp spacer, but most of which are aCa and aCg sites (such as sites 18S-6, 18S-7, 18S-8, 18S-9, 18S-10, 18S-15, and 18S-25 in Figure 5A and 14S-5, 14S-9, 14S-10, and 14S-11 in Figure 5B), which suggested that the effect of nucleotide adjacent to the 3′ end of aC motif also exists for spacers with 18 and 14 base pairs. Meanwhile, it was observed that the DddA-half-dependent editing specificity of G1333 pairs applied equally to the 18bp and the 14bp spacers (Supplementary Figure S1). However, the editing preference of G1397 pairs only could be observed in 18bp spacer, but not in 14bp spacer (Supplementary Figure S1). The detailed editing efficiency of each site is shown in Supplementary Table S7. Taken together, the length of the spacing region only affects the position of editable cytosine for aC targets, while the effect of half preference and nucleotide adjacent to the 3′ end of aC motif also existed for spacers of different length.

### The length of TALEN binding sequence and DddA splitting site influence mtDNA editing specificity

**FIGURE 6:**
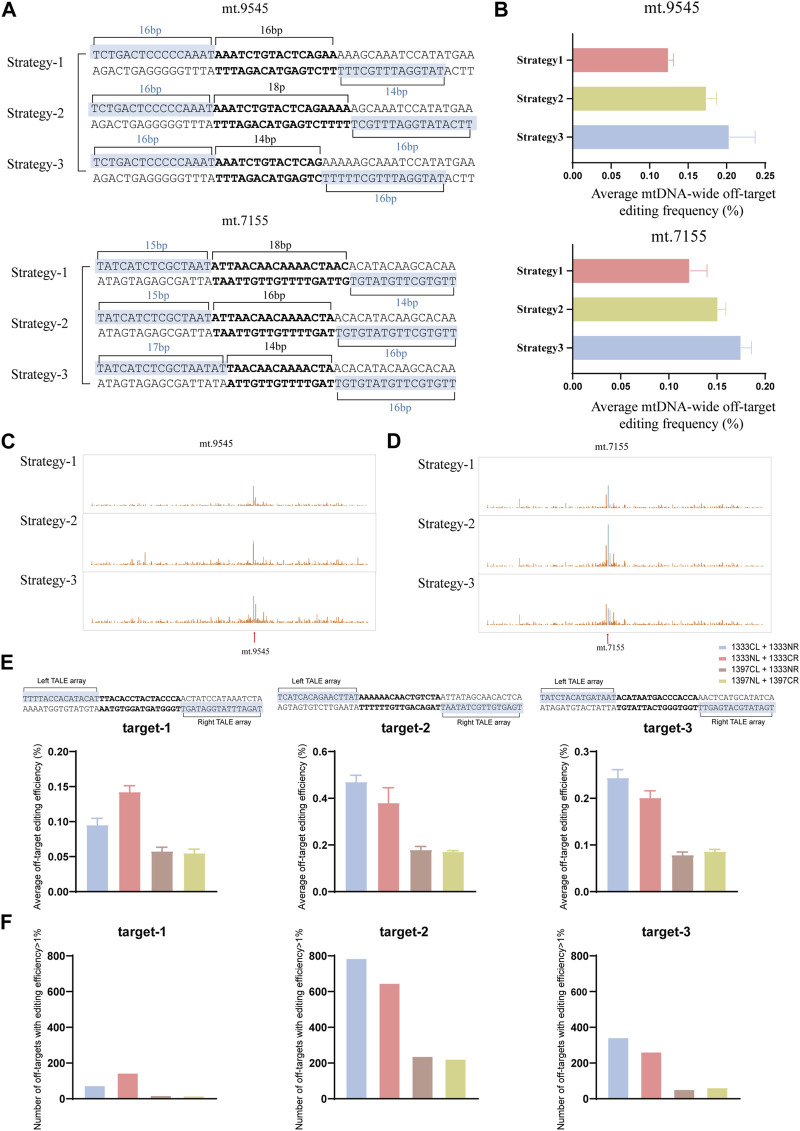
Off-target effects of different TALEN binding sequence and DdCBE pairs. **(A)** The design of mtDNA editing strategy with different TALEN binding sequence and spacer for mt.9545 and mt.7155 sites, respectively. **(B)** Average frequency of mitochonodrial genome-wide C•G-to-T•A off-target editing for each strategy. Values and error bars reflect the mean ± sem of *n* = 3. **(C, D)** The detailed information of mitochondrial genome-wide off-target editing for mt.9545 **(C)** and mt.7155 **(D)**, respectively. For **(C, D)**, the average efficiency of 3 biological replicates was used for analysis. **(E)** Comparisons of average frequency of mitochonodrial genome-wide C•G-to-T•A off-target editing of four DdCBE pairs for each target. Values and error bars reflect the mean ± sem of *n* = 3. **(F)** Comparisons of number of off-target sites (average editing efficiency of 3 biological replicates >1%) of four DdCBE pairs for each target.

It is known that, off-target effects are another factor that affects the application of DdCBEs editors. If there exists more than one option for TALEN binding sequence for mtDNA editing of certain target, it still uncertain how to choose to improve editing specificity. In 2017, Rinaldi and his colleagues used a series of synthetic TALEs to investigate how the number of TALEN RVD repeats affects the its binding affinity to the target and non-target, and the results demonstrated that the specificity (the ratio of affinity for target DNA to affinity for non-target DNA) was variable with increasing number of RVDs, and the optimal length for specificity was between 15 and 19 RVDs (Rinaldi et al., 2017). In this work, to clarify whether the number of RVDs affects the off-target during DdCBE mediated mtDNA editing, two target sites were selected (mt.9545, mt.7155). For each site, three different pairs of TALEN binding sequences (Strategy-1, Strategy-2, and Strategy-3) were named based on the length of the TALEN binding sequence, from short to long, to compare the off-target effects (Figure 6A). To control variables, same split and orientation was used in different strategy (G1333CL+G1333NR for mt.9545, and G1333NL+G1333CR for mt.7155, which was the pair with the highest editing efficiency for each site respectively). As the results indicated, Strategy-1 (TALEN binding sequences of 16bp and 14bp) led to lower average off-target editing frequency than Strategy-2 and 3 (Figure 6B) when three strategies were used for mtDNA editing at mt.9545 site. Similarly, for mt.7155, Strategy-1 (TALEN binding sequences of 15bp and 14bp) led to the lowest average off-target editing frequency (Figure 6B). The detailed information of mitochondrial genome-wide off-targets for this two sites was separately shown in Figures 6C, D. The sequence information of off-target sites of three strategies of mt.9545 and mt.7155 was shown in Supplementary Figure S2. All the off-target sequences (>1%) identified in each strategy were put together to find test sequence similarity. And it was found that there was no similarity between the upstream and downstream sequences of off-target sites and the TALEN recognition sequences of on-target sites. Based on our limited data, it is suspected the number of RVDs may influence mtDNA editing specificity, and sixteen RVDs and above might increase the risk of mitochondrial genome-wide off-targets. This may due to the occurrence of extra binding force caused by excessive RVDs, which might lead to more severe off targets on the entire mitochondrial genome (Rinaldi et al., 2017). However, owing to the limited data, further experiments are needed to verify this suspection.

To explore whether the different DdCBE pair have impact on off-target editing, three target spacers were chosen (target-1, target-2, target-3), and the mitochondrialgenome-wide off-targets among the four pairs of the same spacer were compared. For all the three spacers, G1397 pairs led to lower average off-target editing frequency than G1333 pairs (Figure 6E). And the number of off-targets was also much more in cells edited by G1333 pairs than in cells edited by G1397 pairs (Figure 6F). Thus it is suspected that G1397 pairs have higher editing specificity.

### Construction of a cell model harboring a confirmed pathogenic mtDNA mutation with high accuracy based on the above rules

**FIGURE 7:**
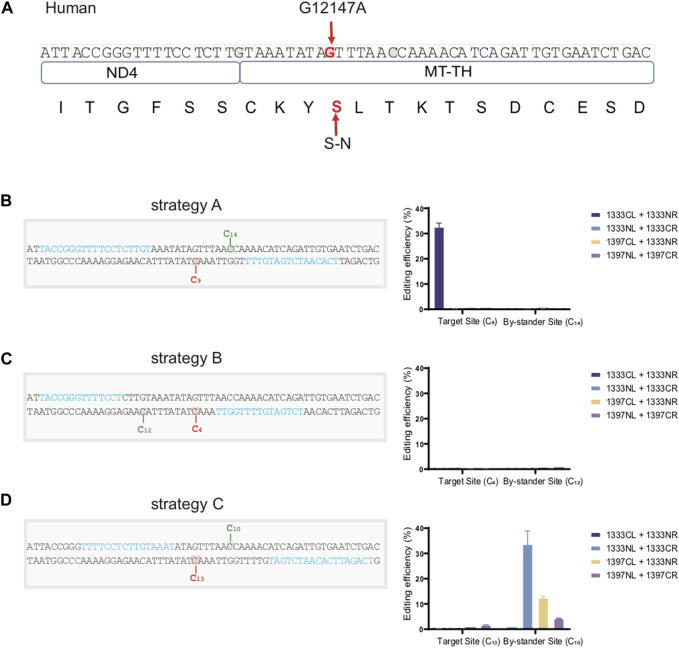
Constructing a cell model containing a pathogenic mutation in mtDNA with high accuracy. **(A)** Schematic overview of the nucleotide sequence and its corresponding amino acid with or without the pathogenic site (mt.12147 G>A) located on the MT-TH gene in human mtDNA. Target amino acid (S) and nucleotide (G) are indicated in the red arrow. **(B)** Information of the spacer and the TALEN target for strategy A, and its corresponding editing efficiency of target and bystander sites with four DdCBE pairs. Target (5′C_9_) and bystander effects sites (5′C_14_) are highlighted in red and green respectively, and the TALE binding sites are indicated in blue. **(C)** Information of the spacer and the TALEN target for strategy B, and its corresponding editing efficiency of target and bystander sites with four DdCBE pairs. Target (5′C_4_) and bystander effects site (5′C_12_) are highlighted in red and green respectively, and the TALE binding sites are indicated in blue. **(D)** Information of the spacer and the TALEN target for strategy C, and its corresponding editing efficiency of target and bystander sites with four DdCBE pairs. Target site (5′C_13_) and bystander effects site (5′C_10_) are highlighted in red and green respectively. TALE binding sites are indicated in blue.

For the convenience for clinical application in the future, we efficiently constructed a cell model containing an identified pathogenic mtDNA mutation located on the MT-TH gene (mt.12147 G>A) under the guidance of the editing rules above. As the mtDNA sequence shown in Figure 7A, besides the target aC motif (marked in red) on the bottom strand, there still exists two additional editable aC sites (marked in green) separately located in the top and bottom strand within the spacing region, which might cause the bystander effects. According to the traditional procedure, a great many time- and effort-consuming screening works need to be performed, including designing different kinds of target spacers and the corresponding four combinations of DddA for each potential target spacer. However, according to our findings above, aC motif on the bottom strand can be effectively edited by the G1333CL+G1333NR pair when the cytosine was put at 5′C_8-10_ in the 16bp spacer. Consequently, we placed the target cytosine at the 5′C_9_ within the 16bp-spacing region (Strategy A). All of the four DdCBE pairs were constructed and editing efficiency was compared (Figure 7B). As expected, only the G1333CL+G1333NR pair could mediate C>T conversion effectively at the mt.12147 site in human U2-OS cells. On the other hand, there was no bystander effect at the potentially editable site (5′C_14_) within the spacer, since this non-target cytosine was out of the editing window 5′C_8-11_ for G1333 splits of DddA. However, another two pairs of TALE arrays were engineered, for which the target cytosine was placed at position at 5′C_4_ and the 5′C_13_ within the spacing region respectively (Strategy B and C), and it was observed that mt.12147 has hardly been edited in four pairs when the other two strategies were used in this work (Figures 7C, D). Remarkably, for strategy C, bystander effects occurred as the non-target cytosine was placed at the position of 5′C_10_, which is within the editing window for both G1333 and G1397 splits of DddA. In summary, the optimal strategy conceived based on our findings did achieve the best editing efficiency without the bystander effects.

## Discussion

**FIGURE 8:**
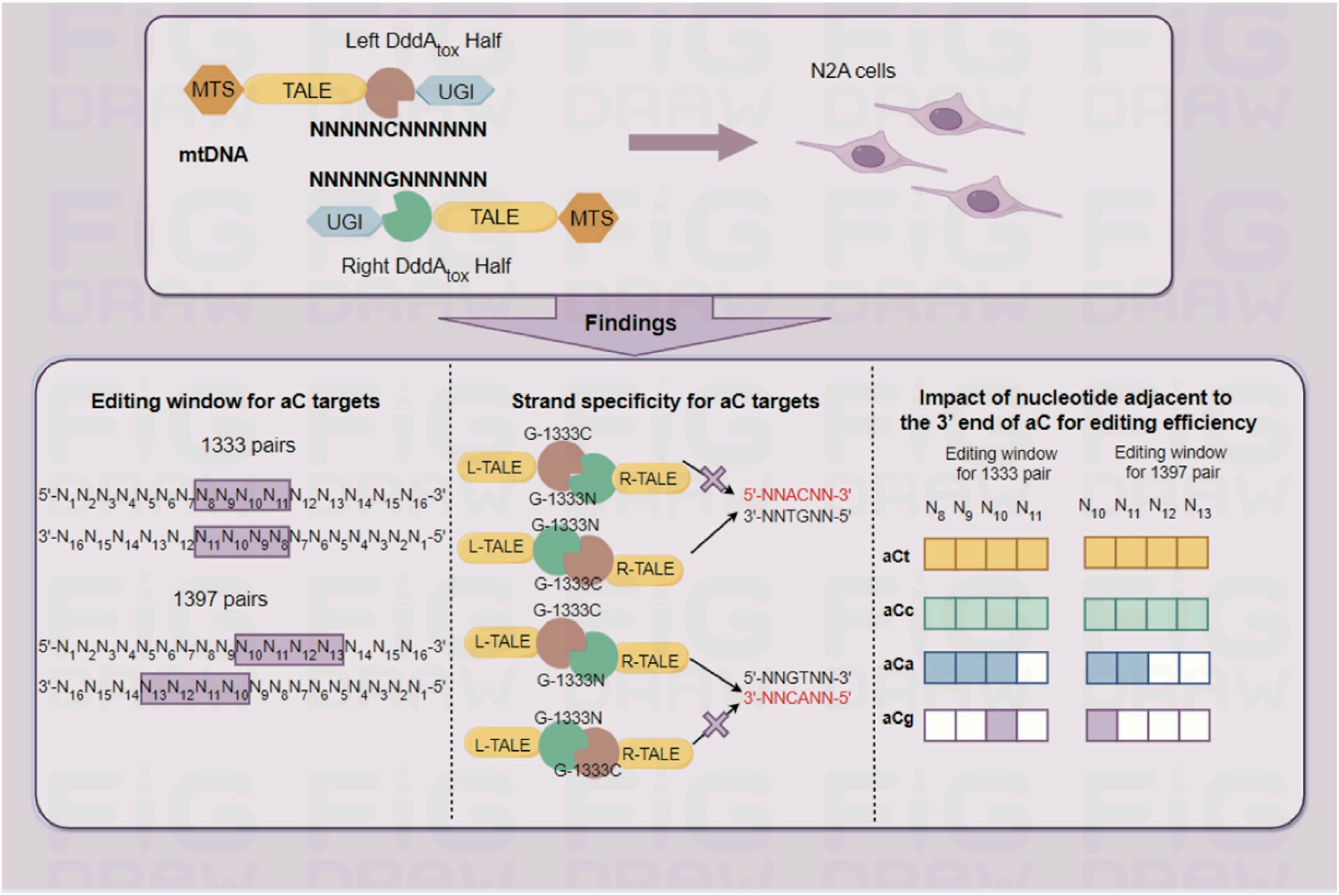
Schematic summary of our findings.

The development of mtDNA editor DdCBE opens the doors for us to explore the pathogenic mechanism of mitochondrial diseases. Meanwhile, it also offers an option for the treatment of relevant mitochondrial disorders in the future. However, how to accurately edit the target nucleotide and reduce bystander effects is a key factor in deciding its potential applicability. To address this problem, strand-selective (Yi et al., 2023) and strand-preferred DddA variants (Hu et al., 2023) have separately been found one after another, aiming at completing strand-specific mtDNA C>T conversion. In this work, it is found that DdCBE also could enable half-specific or half-prefer C>T conversion in mtDNA editing via proper combinations of DddA halves for aC or tC targets. Furthermore, it is also found DdCBEs have some other characterizations for mtDNA editing, for example, aC targets have a more concentrated editing window than tC targets have, and the kind of nucleotide adjacent to the 3′ end of the aC motif affects the efficiency of C>T conversion. All of the above findings were schematically illustrated in Figure 8. This finding will help us perform better in mtDNA editing, making mtDNA editing in a predictable manner.

Based on our findings, DdCBE-mediated mtDNA editing may no longer require tedious screening works to optimize the strategy, the optimal strategy could be designed directly under the guidance of this work. For aC targets with 16 nucleotides in the spacing region, the best position of cytosine is limited to 8–10th nucleotides from the 5′ end of the spacing region. Notably, if the G1397 split of DddA was used for editing aC targets with 16bp spacer, it would be better to put the cytosine at the 10th nucleotide from the 5′ end of the spacing region. Considering the restrictions of TALEN design, there must be target sites located within different lengths of spacers in practice. Thus a series of additional sites within 18bp and 14bp spacer were selected, providing some references for future research. For spacers with different lengths, the editing window of G1333 pair appears to locate in the middle of the spacer according to the spacer length (5′ C_7-10_ for 14bp spacer; 5′ C_8-11_ for 16bp spacer; 5′ C_7-13_ for 18bp spacer), while the editing window of G1397 pair is biased towards the 3′ end.

Moreover, it is suggested that G1333N split of DddA should be designed to bind the strand containing target aC because G1333 splits of DddA only could catalyze cytosine located on the N terminal targeting strand, while aC on the other strand is hard to edit. So it is suggested to use G1333 pairs for aC editing when attempting to avoid unwarranted bystander editing on the other strand. As the priority editing site of DdCBE, tC editing seems to have less restrictions than aC targets. However, editing preferences still existed between different orientations. For tC on the top strand, G1333NL+G1333CR pair were suggested for higher editing efficiency in most cases; while G1333CL+G1333NR pair was suggested for tC on the bottom strand for effective editing. When tC is located on the left half of the spacer, G1397NL+G1397CR pair can achieve better editing results, while the other pair should be chosen when tC is located on the right half of the spacer, although the disparity of editing efficiency was not obvious between the two orientations of G1397 pair.

Furthermore, since the kind of nucleotide adjacent to the 3′ end of aC motif also impacts the efficiency of C>T conversion, special attention should be paid when the target site is aCg - it can only be edited when the cytosine was put at the 5′C_10_ for both G1333 and G1397 splits of DddA. Also, if a mitochondrial gene with an aCa motif is selected as a target, it would be better to perform the mtDNA editing with the G1333 splits, since the targets with aCa motif can only be edited with low efficiency by G1397 splits, and that’s why some aCa targets could not be effectively edited by G1397 splits in our work, even though the cytosine was put at the editing window. Remarkably, the influence of the 3′ end nucleotide adjacent to the aC motif and strand bias of the editor still exists in the spacer with 18 or 14 base pairs in length.

Taken together, by screening the combination of different DdCBE pairs for a wide range of tested mtDNA targets, it is found that DdCBE edits mtDNA are predictable. This finding will guide us to perform better in relevant mtDNA editing. Firstly, it helps us select suitable spacer and editor pairs for target sites, effectively saving time and effort to optimize the strategy. On the other hand, it allows us to use the strand dependency rules to eliminate unnecessary bystander effects, aiming for precise editing in repairing the mtDNA point mutations in mitochondrial diseases.

However, the conclusions only have been obtained based on less than 100 target sites, and have only been validated at one mitochondrial disease-relevant site in our work, more targets need to be selected to confirm our results in the future. On the other hand, genome-wide off-target editing is another factor either in mitochondrial genome or in the nuclear genome. The nuclear off-target effects of DdCBE system have been reported in cells (Lei et al., 2022), embryos (Wei et al., 2022b), and mice (Lee et al., 2022b). There are three different strategies to prevent nuclear off-target editing by DdCBEs: (1) adding nuclear export signal (NES) sequences to the DdCBE to reduce nuclear localization of the DdCBE protein - it can not only significantly reduce nuclear off-targets, but can also achieve more efficient editing of the mtDNA on-target; (2) simultaneously expressing DddIA (a naturally occurring inhibitor of the deaminase DddA) fused to a bipartite nuclear localization signal to antagonize the nuclear editing activity of DdCBE; (3) mutating DddA_tox_ to decrease its spontaneous assembly, including HIFI-DdCBE which substitutes alanine for amino acid residues at the interface between the split DddA_tox_ halves (Lee et al., 2022a).

## Data Availability

The datasets presented in this study can be found in online repositories. The names of the repository/repositories and accession number(s) can be found in the article/Supplementary Material.

## Supplementary material

SUPPLEMENTARY FIGURE S1 Comparison of the editing efficiency of targets with aC motif on the top and bottom strand within 18bp and 14bp spacer. **(A, B)** Comparison of editing efficiency of aC targets on the top strand **(A)** and bottom strand **(B)** within 18bp spacer using G1333 splits with different orientation. **(C, D)** Comparison of editing efficiency of aC targets on the top strand **(C)** and bottom strand **(D)** within 18bp spacer using G1397 splits with different orientation. **(E, F)** Comparisons of editing efficiency of aC targets on the top strand **(E)** and bottom strand **(F)** within 14bp spacer using G1333 splits with different orientation. **(G)** Comparison of editing efficiency of aC targets on the top strand within 14bp spacer using G1397 splits with different orientation. Data were represented as mean ± sem, and the number of biological replicates is at least three time for each site.

SUPPLEMENTARY FIGURE S2 The sequence information of off-target sites of three strategies for mt.9545 **(A)** and mt.7155 **(B)**, respectively. The left and right TALE binding sequences were marked with blue shaded areas respectively.

SUPPLEMENTARY TABLE S1 Amino acids sequence of DdCBEs used in this study.

SUPPLEMENTARY TABLE S2 Primers used in this study.

SUPPLEMENTARY TABLE S3 Spacer sequence, mitoTALE target sequence, and site locus for each target.

SUPPLEMENTARY TABLE S4 Next-generation sequencing results of 3 biological replicates per DdCBE pair of aC targets.

SUPPLEMENTARY TABLE S5 Next-generation sequencing results of 3 biological replicates per DdCBE pair of tC targets.

SUPPLEMENTARY TABLE S6 The specific editor combination used for each site in Figure 1.

SUPPLEMENTARY TABLE S7 Next-generation sequencing results of 3 biological replicates per DdCBE pair of aC targets within 18bp and 14bp spacer.

## References

[B1] Allen F. Crepaldi L. Alsinet C. Strong A. J. Kleshchevnikov V. De Angeli P. (2019). Predicting the mutations generated by repair of Cas9-induced double-strand breaks. Nat. Biotechnol. 37, 64–72. 10.1038/nbt.4317 PMC6949135 30480667

[B2] Arabi F. Mansouri V. Ahmadbeigi N. (2022). Gene therapy clinical trials, where do we go? An overview. Biomed. Pharmacother. = Biomedecine Pharmacother. 153, 113324. 10.1016/j.biopha.2022.113324 35779421

[B3] Bacman S. R. Kauppila J. H. K. Pereira C. V. Nissanka N. Miranda M. Pinto M. (2018). MitoTALEN reduces mutant mtDNA load and restores tRNAAla levels in a mouse model of heteroplasmic mtDNA mutation. Nat. Med. 24, 1696–1700. 10.1038/s41591-018-0166-8 30250143 PMC6942693

[B4] Bacman S. R. Williams S. L. Pinto M. Peralta S. Moraes C. T. (2013). Specific elimination of mutant mitochondrial genomes in patient-derived cells by mitoTALENs. Nat. Med. 19, 1111–1113. 10.1038/nm.3261 23913125 PMC4153471

[B5] Boyne A. Yang M. Pulicani S. Feola M. Tkach D. Hong R. (2022). Efficient multitool/multiplex gene engineering with TALE-BE. Front. Bioeng. Biotechnol. 10, 1033669. 10.3389/fbioe.2022.1033669 36440442 PMC9684181

[B6] Chakrabarti A. M. Henser-Brownhill T. Monserrat J. Poetsch A. R. Luscombe N. M. Scaffidi P. (2019). Target-specific precision of CRISPR-mediated genome editing. Mol. Cell. 73, 699–713.e6. 10.1016/j.molcel.2018.11.031 30554945 PMC6395888

[B7] Chen B. S. Yu-Wai-Man P. (2022). From bench to bedside-delivering gene therapy for leber hereditary optic neuropathy. Cold Spring Harb. Perspect. Med. 12, a041282. 10.1101/cshperspect.a041282 35863905 PMC9310952

[B8] Chen W. McKenna A. Schreiber J. Haeussler M. Yin Y. Agarwal V. (2019). Massively parallel profiling and predictive modeling of the outcomes of CRISPR/Cas9-mediated double-strand break repair. Nucleic acids Res. 47, 7989–8003. 10.1093/nar/gkz487 31165867 PMC6735782

[B9] Chen X. Liang D. Guo J. Zhang J. Sun H. Zhang X. (2022). DdCBE-mediated mitochondrial base editing in human 3PN embryos. Cell. Discov. 8, 8. 10.1038/s41421-021-00358-y 35102135 PMC8803914

[B10] Chinnery P. F. (2015). Mitochondrial disease in adults: what's old and what's new? EMBO Mol. Med. 7, 1503–1512. 10.15252/emmm.201505079 26612854 PMC4693502

[B11] Cho S. I. Lee S. Mok Y. G. Lim K. Lee J. Lee J. M. (2022). Targeted A-to-G base editing in human mitochondrial DNA with programmable deaminases. Cell. 185, 1764–1776.e12. 10.1016/j.cell.2022.03.039 35472302

[B12] De Ravin S. S. Li L. Wu X. Choi U. Allen C. Koontz S. (2017). CRISPR-Cas9 gene repair of hematopoietic stem cells from patients with X-linked chronic granulomatous disease. Sci. Transl. Med. 9, eaah3480. 10.1126/scitranslmed.aah3480 28077679

[B13] Gammage P. A. Gaude E. Van Haute L. Rebelo-Guiomar P. Jackson C. B. Rorbach J. (2016a). Near-complete elimination of mutant mtDNA by iterative or dynamic dose-controlled treatment with mtZFNs. Nucleic acids Res. 44, 7804–7816. 10.1093/nar/gkw676 27466392 PMC5027515

[B14] Gammage P. A. Moraes C. T. Minczuk M. (2018a). Mitochondrial genome engineering: the revolution may not Be CRISPR-ized. Trends Genet. 34, 101–110. 10.1016/j.tig.2017.11.001 29179920 PMC5783712

[B15] Gammage P. A. Rorbach J. Vincent A. I. Rebar E. J. Minczuk M. (2014). Mitochondrially targeted ZFNs for selective degradation of pathogenic mitochondrial genomes bearing large-scale deletions or point mutations. EMBO Mol. Med. 6, 458–466. 10.1002/emmm.201303672 24567072 PMC3992073

[B16] Gammage P. A. Van Haute L. Minczuk M. (2016b). Engineered mtZFNs for manipulation of human mitochondrial DNA heteroplasmy. Methods Mol. Biol. 1351, 145–162. 10.1007/978-1-4939-3040-1_11 26530680

[B17] Gammage P. A. Viscomi C. Simard M. L. Costa A. S. H. Gaude E. Powell C. A. (2018b). Genome editing in mitochondria corrects a pathogenic mtDNA mutation *in vivo* . Nat. Med. 24, 1691–1695. 10.1038/s41591-018-0165-9 30250142 PMC6225988

[B18] Gorman G. S. Chinnery P. F. DiMauro S. Hirano M. Koga Y. McFarland R. (2016). Mitochondrial diseases. Nat. Rev. Dis. Prim. 2, 16080. 10.1038/nrdp.2016.80 27775730

[B19] Grady J. P. Pickett S. J. Ng Y. S. Alston C. L. Blakely E. L. Hardy S. A. (2018). mtDNA heteroplasmy level and copy number indicate disease burden in m.3243A>G mitochondrial disease. EMBO Mol. Med. 10, e8262. 10.15252/emmm.201708262 29735722 PMC5991564

[B20] Greenfield A. Braude P. Flinter F. Lovell-Badge R. Ogilvie C. Perry A. C. F. (2017). Assisted reproductive technologies to prevent human mitochondrial disease transmission. Nat. Biotechnol. 35, 1059–1068. 10.1038/nbt.3997 29121011

[B21] Guo J. Chen X. Liu Z. Sun H. Zhou Y. Dai Y. (2022). DdCBE mediates efficient and inheritable modifications in mouse mitochondrial genome. Mol. Ther. Nucleic acids 27, 73–80. 10.1016/j.omtn.2021.11.016 34938607 PMC8646052

[B22] Guo J. Yu W. Li M. Chen H. Liu J. Xue X. (2023). A DddA ortholog-based and transactivator-assisted nuclear and mitochondrial cytosine base editors with expanded target compatibility. Mol. Cell. 83, 1710–1724.e7. 10.1016/j.molcel.2023.04.012 37141888

[B23] Guo J. Zhang X. Chen X. Sun H. Dai Y. Wang J. (2021). Precision modeling of mitochondrial diseases in zebrafish via DdCBE-mediated mtDNA base editing. Cell. Discov. 7, 78. 10.1038/s41421-021-00307-9 34480028 PMC8417030

[B24] Hu J. Sun Y. Li B. Liu Z. Wang Z. Gao Q. (2023). Strand-preferred base editing of organellar and nuclear genomes using CyDENT. Nat. Biotechnol. 10.1038/s41587-023-01910-9 37640945

[B25] Kang B. C. Bae S. J. Lee S. Lee J. S. Kim A. Lee H. (2021). Chloroplast and mitochondrial DNA editing in plants. Nat. plants 7, 899–905. 10.1038/s41477-021-00943-9 34211132 PMC8289734

[B26] Lee H. Lee S. Baek G. Kim A. Kang B.-C. Seo H. (2021). Mitochondrial DNA editing in mice with DddA-TALE fusion deaminases. Nat. Commun. 12, 1190. 10.1038/s41467-021-21464-1 33608520 PMC7895935

[B27] Lee S. Lee H. Baek G. Kim J.-S. (2022a). Precision mitochondrial DNA editing with high-fidelity DddA-derived base editors. Nat. Biotechnol. 41, 378–386. 10.1038/s41587-022-01486-w 36229610 PMC10017512

[B63] Lee H. Baek G. Namgung E. Park J. M. Kim S. Hong S. (2022b). Enhanced mitochondrial DNA editing in mice using nuclear-exported TALE-linked deaminases and nucleases. Genome biology 23, 211. 10.1186/s13059-022-02782-z 36224582 PMC9554978

[B64] Lei Z. Meng H. Liu L. Zhao H. Rao X. Yan Y. (2022). Mitochondrial base editor induces substantial nuclear off-target mutations. Nature 606, 804–811. 10.1038/s41586-022-04836-5 35551512

[B28] Ling Q. Rioux M. Hu Y. Lee M. Gray S. (2021). Adeno-associated viral vector serotype 9-based gene replacement therapy for SURF1-related Leigh syndrome. Mol. Ther. Methods Clin. Dev. 23, 158–168. 10.1016/j.omtm.2021.09.001 34703839 PMC8517205

[B29] Long C. (2019). God does not play dice, and neither does CRISPR/Cas9. Natl. Sci. Rev. 6, 393. 10.1093/nsr/nwy156 34691887 PMC8291601

[B30] McFarland R. Taylor R. W. Turnbull D. M. (2010). A neurological perspective on mitochondrial disease. Lancet. Neurology 9, 829–840. 10.1016/s1474-4422(10)70116-2 20650404

[B31] Meng F. Jia Z. Zheng J. Ji Y. Wang J. Xiao Y. (2022). A deafness-associated mitochondrial DNA mutation caused pleiotropic effects on DNA replication and tRNA metabolism. Nucleic acids Res. 50, 9453–9469. 10.1093/nar/gkac720 36039763 PMC9458427

[B32] Mi L. Shi M. Li Y. X. Xie G. Rao X. Wu D. (2023). DddA homolog search and engineering expand sequence compatibility of mitochondrial base editing. Nat. Commun. 14, 874. 10.1038/s41467-023-36600-2 36797253 PMC9935910

[B33] Minczuk M. Papworth M. A. Miller J. C. Murphy M. P. Klug A. (2008). Development of a single-chain, quasi-dimeric zinc-finger nuclease for the selective degradation of mutated human mitochondrial DNA. Nucleic acids Res. 36, 3926–3938. 10.1093/nar/gkn313 18511461 PMC2475635

[B34] Mok B. Y. de Moraes M. H. Zeng J. Bosch D. E. Kotrys A. V. Raguram A. (2020). A bacterial cytidine deaminase toxin enables CRISPR-free mitochondrial base editing. Nature 583, 631–637. 10.1038/s41586-020-2477-4 32641830 PMC7381381

[B35] Mok B. Y. Kotrys A. V. Raguram A. Huang T. P. Mootha V. K. Liu D. R. (2022). CRISPR-free base editors with enhanced activity and expanded targeting scope in mitochondrial and nuclear DNA. Nat. Biotechnol. 40, 1378–1387. 10.1038/s41587-022-01256-8 35379961 PMC9463067

[B36] Nelson C. E. Hakim C. H. Ousterout D. G. Thakore P. I. Moreb E. A. Castellanos Rivera R. M. (2016). *In vivo* genome editing improves muscle function in a mouse model of Duchenne muscular dystrophy. Sci. (New York, N.Y.) 351, 403–407. 10.1126/science.aad5143 PMC4883596 26721684

[B37] Nissanka N. Bacman S. R. Plastini M. J. Moraes C. T. (2018). The mitochondrial DNA polymerase gamma degrades linear DNA fragments precluding the formation of deletions. Nat. Commun. 9, 2491. 10.1038/s41467-018-04895-1 29950568 PMC6021392

[B38] Peeva V. Blei D. Trombly G. Corsi S. Szukszto M. J. Rebelo-Guiomar P. (2018). Linear mitochondrial DNA is rapidly degraded by components of the replication machinery. Nat. Commun. 9, 1727. 10.1038/s41467-018-04131-w 29712893 PMC5928156

[B39] Pereira C. V. Bacman S. R. Arguello T. Zekonyte U. Williams S. L. Edgell D. R. (2018). mitoTev-TALE: a monomeric DNA editing enzyme to reduce mutant mitochondrial DNA levels. EMBO Mol. Med. 10, e8084. 10.15252/emmm.201708084 30012581 PMC6127889

[B40] Qi X. Chen X. Guo J. Zhang X. Sun H. Wang J. (2021). Precision modeling of mitochondrial disease in rats via DdCBE-mediated mtDNA editing. Cell. Discov. 7, 95. 10.1038/s41421-021-00325-7 34663794 PMC8523528

[B41] Qi X. Tan L. Zhang X. Jin J. Kong W. Chen W. (2023). Expanding DdCBE-mediated targeting scope to aC motif preference in rat. Nucleic acids. 32, 1–12. 10.1016/j.omtn.2023.02.028 36942261 PMC10023868

[B42] Reddy P. Ocampo A. Suzuki K. Luo J. Bacman S. R. Williams S. L. (2015). Selective elimination of mitochondrial mutations in the germline by genome editing. Cell. 161, 459–469. 10.1016/j.cell.2015.03.051 25910206 PMC4505837

[B43] Rinaldi F. C. Doyle L. A. Stoddard B. L. Bogdanove A. J. (2017). The effect of increasing numbers of repeats on TAL effector DNA binding specificity. Nucleic acids Res. 45, 6960–6970. 10.1093/nar/gkx342 28460076 PMC5499867

[B44] Russell O. M. Gorman G. S. Lightowlers R. N. Turnbull D. M. (2020). Mitochondrial diseases: hope for the future. Cell. 181, 168–188. 10.1016/j.cell.2020.02.051 32220313

[B45] Schapira A. H. (2012). Mitochondrial diseases. Lancet London, Engl. 379, 1825–1834. 10.1016/s0140-6736(11)61305-6 22482939

[B46] Sharma R. Anguela X. M. Doyon Y. Wechsler T. DeKelver R. C. Sproul S. (2015). *In vivo* genome editing of the albumin locus as a platform for protein replacement therapy. Blood 126, 1777–1784. 10.1182/blood-2014-12-615492 26297739 PMC4600017

[B47] Shen M. W. Arbab M. Hsu J. Y. Worstell D. Culbertson S. J. Krabbe O. (2018). Predictable and precise template-free CRISPR editing of pathogenic variants. Nature 563, 646–651. 10.1038/s41586-018-0686-x 30405244 PMC6517069

[B48] Shi X. Shou J. Mehryar M. M. Li J. Wang L. Zhang M. (2019). Cas9 has no exonuclease activity resulting in staggered cleavage with overhangs and predictable di- and tri-nucleotide CRISPR insertions without template donor. Cell. Discov. 5, 53. 10.1038/s41421-019-0120-z 31636963 PMC6796948

[B49] Shou J. Li J. Liu Y. Wu Q. (2018). Precise and predictable CRISPR chromosomal rearrangements reveal principles of cas9-mediated nucleotide insertion. Mol. Cell. 71, 498–509.e4. 10.1016/j.molcel.2018.06.021 30033371

[B50] Silva-Pinheiro P. Cerutti R. Luna-Sanchez M. Zeviani M. Viscomi C. (2020). A single intravenous injection of AAV-PHP.B-hNDUFS4 ameliorates the phenotype of Ndufs4−/−mice. Mol. Ther. Methods Clin. Dev. 17, 1071–1078. 10.1016/j.omtm.2020.04.026 32478122 PMC7248291

[B51] Silva-Pinheiro P. Minczuk M. (2022). The potential of mitochondrial genome engineering. Nat. Rev. Genet. 23, 199–214. 10.1038/s41576-021-00432-x 34857922

[B52] Silva-Pinheiro P. Mutti C. D. Van Haute L. Powell C. A. Nash P. A. Turner K. (2023). A library of base editors for the precise ablation of all protein-coding genes in the mouse mitochondrial genome. Nat. Biomed. Eng. 7, 692–703. 10.1038/s41551-022-00968-1 36470976 PMC10195678

[B53] Silva-Pinheiro P. Nash P. A. Van Haute L. Mutti C. D. Turner K. Minczuk M. (2022). *In vivo* mitochondrial base editing via adeno-associated viral delivery to mouse post-mitotic tissue. Nat. Commun. 13, 750. 10.1038/s41467-022-28358-w 35136065 PMC8825850

[B54] van Overbeek M. Capurso D. Carter M. M. Thompson M. S. Frias E. Russ C. (2016). DNA repair profiling reveals nonrandom outcomes at cas9-mediated breaks. Mol. Cell. 63, 633–646. 10.1016/j.molcel.2016.06.037 27499295

[B55] Viscomi C. van den Ameele J. Meyer K. C. Chinnery P. F. (2023). Opportunities for mitochondrial disease gene therapy. Nat. Rev. Drug Discov. 22, 429–430. 10.1038/d41573-023-00067-z 37106085

[B56] Wei Y. Jin M. Huang S. Yao F. Ren N. Xu K. (2023). Enhanced C-To-T and A-to-G base editing in mitochondrial DNA with engineered DdCBE and TALED. Adv. Sci. Weinheim, Baden-Wurttemberg, Ger. 11, e2304113. 10.1002/advs.202304113 PMC10797475 37984866

[B57] Wei Y. Li Z. Xu K. Feng H. Xie L. Li D. (2022a). Mitochondrial base editor DdCBE causes substantial DNA off-target editing in nuclear genome of embryos. Cell. Discov. 8, 27. 10.1038/s41421-022-00391-5 35304438 PMC8933521

[B58] Wei Y. Xu C. Feng H. Xu K. Li Z. Hu J. (2022b). Human cleaving embryos enable efficient mitochondrial base-editing with DdCBE. Cell. Discov. 8, 7. 10.1038/s41421-021-00372-0 35102133 PMC8803867

[B59] wLee S. Lee H. Baek G. Namgung E. Park J. M. Kim S. (2022b). Enhanced mitochondrial DNA editing in mice using nuclear-exported TALE-linked deaminases and nucleases. Genome Biol. 23, 211. 10.1186/s13059-022-02782-z 36224582 PMC9554978

[B60] Yang Y. Wu H. Kang X. Liang Y. Lan T. Li T. (2018). Targeted elimination of mutant mitochondrial DNA in MELAS-iPSCs by mitoTALENs. Protein & Cell. 9, 283–297. 10.1007/s13238-017-0499-y 29318513 PMC5829275

[B61] Yi Z. Zhang X. Tang W. Yu Y. Wei X. Zhang X. (2023). Strand-selective base editing of human mitochondrial DNA using mitoBEs. Nat. Biotechnol. 42, 498–509. Online ahead of print. 10.1038/s41587-023-01791-y 37217751 PMC10940147

[B62] Zheng J. Suo L. Zhou Y. Jia L. Li J. Kuang Y. (2022). Pyk2 suppresses contextual fear memory in an autophosphorylation-independent manner. J. Mol. Cell. Biol. 13, 808–821. 10.1093/jmcb/mjab057 34529077 PMC8782590

