# Supplementary figures and images for: Harnessing accurate mitochondrial DNA base editing mediated by DdCBEs in a predictable manner
Source: Front Bioeng Biotechnol. 2024 Apr 9;12:1372211. doi: 10.3389/fbioe.2024.1372211 (PMC11035818; doi:10.3389/fbioe.2024.1372211)

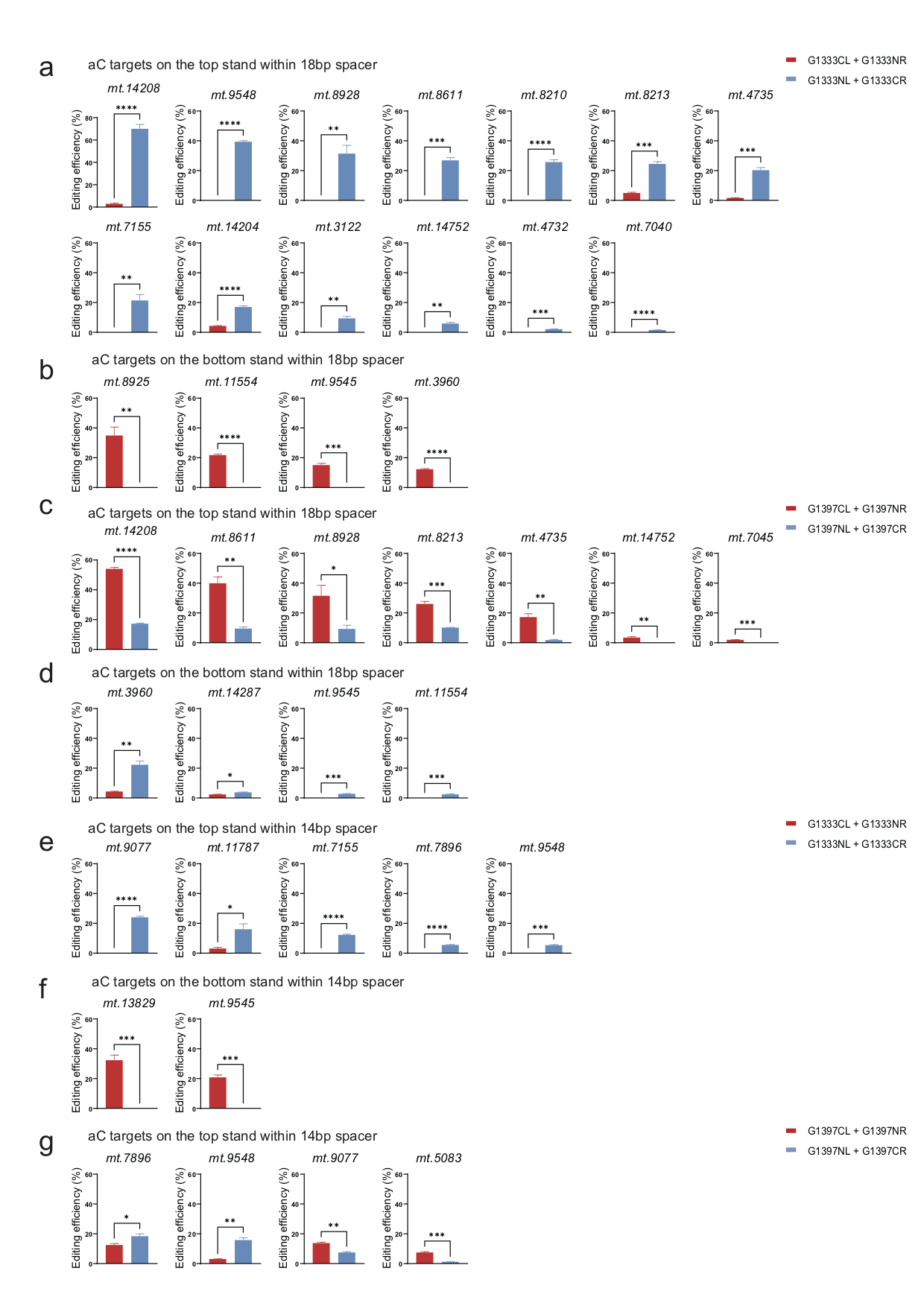

Supplement: Supplementary file 2 [file Image1.TIFF]

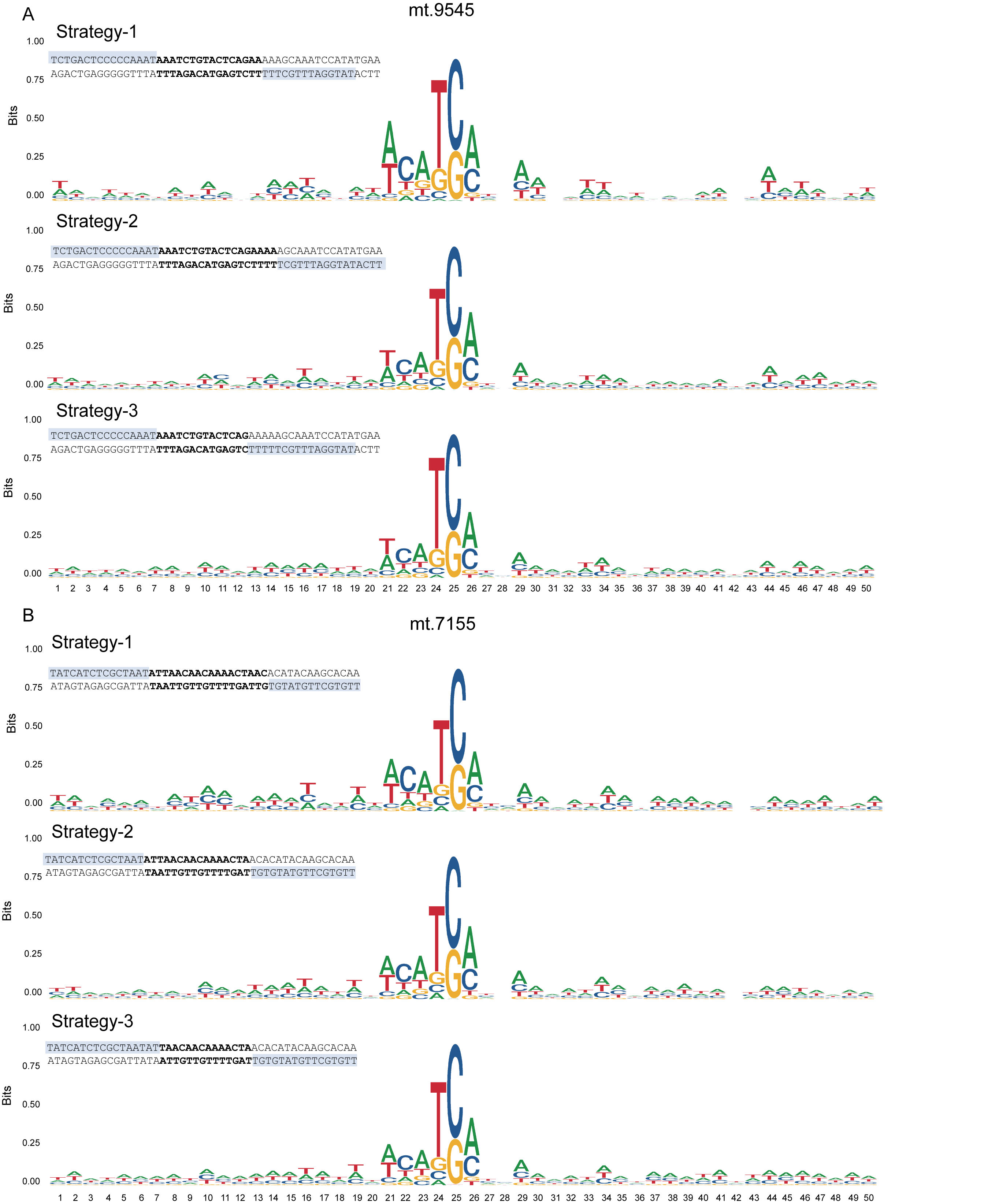

Supplement: Supplementary file 4 [file Image2.TIF]
